# Supplementary material for: Economic evaluation of hypertension screening in Iran using a Markov model
Source: PLoS One. 2025 Jul 22;20(7):e0303223. doi: 10.1371/journal.pone.0303223 (PMC12282904; doi:10.1371/journal.pone.0303223)
Supplement: S1 Appendix — (DOCX) [file pone.0303223.s001.docx]

Appendix: Some possibilities used in the model

| Age groups | Probability of non-cardiovascular death(NCD) | The possibility of cardiovascular death(CV) | Probability of transition from healthy state to CHD | Possibility of high blood pressure | Probability of transition from healthy state to stroke | Probability of transition from untreated hypertension to CHD event | Probability of transition from untreated hypertension to stroke event | Probability of receiving treatment by people with high blood pressure |
| --- | --- | --- | --- | --- | --- | --- | --- | --- |
| 0-4 | 0.001987 | 0000127442 | 0 | 0 | 0 | 0 | 0 | 0 |
| 5-9 | 0.001986 | 0.0000142013 | 0 | 0 | 0 | 0 | 0 | 0 |
| 10-14 | 0.001979 | 0.0000210511 | 0 | 0 | 0 | 0 | 0 | 0 |
| 15-19 | 0.003939 | 0.0000608688 | 0 | 0.0169386 | 0 | 0 | 0 | 0.028341 |
| 20-24 | 0.004923 | 0.0000769166 | 0 | 0.0169386 | 0 | 0 | 0 | 0.028341 |
| 25-29 | 0.0049 | 0.0000996996 | 0 | 0.0131742 | 0 | 0 | 0 | 0.092507 |
| 30-34 | 0.004851 | 0.0001488604 | 0.00098 | 0.0131742 | 0.000388 | 0.00182 | 0.001079 | 0.092507 |
| 35-39 | 0.005762 | 0.0002376375 | 0.00139 | 0.0163457 | 0.000513 | 0.00257 | 0.001427 | 0.149579 |
| 40-44 | 0.007574 | 0.0004264548 | 0.00285 | 0.0163457 | 0.000678 | 0.00527 | 0.001883 | 0.149579 |
| 45-49 | 0.01121 | 0.0007900792 | 0.00514 | 0.0126266 | 0.000943 | 0.0095 | 0.002621 | 0.079561 |
| 50-54 | 0.017517 | 0.0014828892 | 0.00978 | 0.0126266 | 0.001447 | 0.01802 | 0.004019 | 0.079561 |
| 55-59 | 0.027378 | 0.0026216460 | 0.01614 | 0.0182840 | 0.00188 | 0.02969 | 0.005218 | 0.047164 |
| 60-64 | 0.044508 | 0.0044921111 | 0.02158 | 0.0182840 | 0.002221 | 0.03957 | 0.00616 | 0.047164 |
| 65-69 | 0.068656 | 0.0073435881 | 0.02654 | 0.0246800 | 0.002716 | 0.04857 | 0.007531 | 0.001034 |
| 70-74 | 0.107389 | 0.0126110473 | 0.03122 | 0.0246800 | 0.003073 | 0.05698 | 0.008518 | 0.001034 |
| 75-79 | 0.184854 | 0.0251455209 | 0.03406 | 0.0077568 | 0.003993 | 0.06199 | 0.011061 | 0.178561 |
| 80+ | 0.268363 | 0.0616372284 | 0.04927 | 0.0077568 | 0.005592 | 0.08911 | 0.015466 | 0.178561 |
