# Supplementary material for: Economic evaluation of hypertension screening in Iran using a Markov model
Source: PLoS One. 2025 Jul 22;20(7):e0303223. doi: 10.1371/journal.pone.0303223 (PMC12282904; doi:10.1371/journal.pone.0303223)
Supplement: S1 Checklist — (DOCX) [file pone.0303223.s002.docx]

CHEERS 2022 Checklist

|  | **Item** | **Guidance for Reporting** | **Reported in section** |
| --- | --- | --- | --- |
| **TITLE** |  |  |  |
| TITLE | 1 | Identify the study as an economic evaluation and specify the interventions being compared. | Yes, this is clearly stated in both the title and the abstract method section. |
| **Abstract** |  |  |  |
| Abstract | 2 | Provide a structured summary that highlights context, key methods, results and alternative analyses. | These elements are clearly specified in the abstract. |
| **INTRODUCTION** |  |  |  |
| Background and objectives | 3 | Give the context for the study, the study question and its practical relevance for decision making in policy or practice. | This is clearly stated in the introduction section of the paper. |
| **METHODS** |  |  |  |
| Health economic analysis plan | 4 | Indicate whether a health economic analysis plan was developed and where available. | Yes, it is stated at the beginning of the methods section. |
| Study population | 5 | Describe characteristics of the study population (such as age range, demographics, socioeconomic, or clinical characteristics). | Yes, these characteristics are fully defined in the study and model. |
| Setting and location | 6 | Provide relevant contextual information that may influence findings. | All relevant contextual factors are referenced in the tables and text of the methods section. |
| Comparators | 7 | Describe the interventions or strategies being compared and why chosen. | These are described in detail in the methods section. |
| Perspective | 8 | State the perspective(s) adopted by the study and why chosen. | The study's perspective is addressed. |
| Time horizon | 9 | State the time horizon for the study and why appropriate. | This is clearly stated. |
| Discount rate | 10 | Report the discount rate(s) and reason chosen. | The discount rate is specified. |
| Selection of outcomes | 11 | Describe what outcomes were used as the measure(s) of benefit(s) and harm(s). | These are clearly stated. |
| Measurement of outcomes | 12 | Describe how outcomes used to capture benefit(s) and harm(s) were measured. | The outcomes are measured using modeling. |
| Valuation of outcomes | 13 | Describe the population and methods used to measure and value outcomes. | This is specified. |
| Measurement and valuation of resources and costs | 14 | Describe how costs were valued. | This is indicated in the tables and text. |
| Currency, price date, and conversion | 15 | Report the dates of the estimated resource quantities and unit costs, plus the currency and year of conversion. | These details are provided in the study. |
| Rationale and description of model | 16 | If modelling is used, describe in detail and why used. Report if the model is publicly available and where it can be accessed. | All these aspects are addressed in the methods section. |
| Analytics and assumptions | 17 | Describe any methods for analysing or statistically transforming data, any extrapolation methods, and approaches for validating any model used. | Modeling is used, and model validation is confirmed in the studies referenced. |
| Characterizing heterogeneity | 18 | Describe any methods used for estimating how the results of the study vary for sub-groups. | These aspects are addressed with modeling. |
| Characterizing distributional effects | 19 | Describe how impacts are distributed across different individuals or adjustments made to reflect priority populations. | These aspects are also addressed with modeling. |
| Characterizing uncertainty | 20 | Describe methods to characterize any sources of uncertainty in the analysis. | Sensitivity analysis is conducted. |
| Approach to engagement with patients and others affected by the study | 21 | Describe any approaches to engage patients or service recipients, the general public, communities, or stakeholders (e.g., clinicians or payers) in the design of the study. | For this study, there was no direct engagement with patients, and data-related aspects are mentioned in the article. |
| **RESULTS** |  |  |  |
| Study parameters | 22 | Report all analytic inputs (e.g., values, ranges, references) including uncertainty or distributional assumptions. | These are addressed in the tables and findings. |
| Summary of main results | 23 | Report the mean values for the main categories of costs and outcomes of interest and summarise them in the most appropriate overall measure. | These are reported in the findings. |
| Effect of uncertainty | 24 | Describe how uncertainty about analytic judgments, inputs, or projections affect findings. Report the effect of choice of discount rate and time horizon, if applicable. | This is addressed in the findings. |
| Effect of engagement with patients and others affected by the study | 25 | Report on any difference patient/service recipient, general public, community, or stakeholder involvement made to the approach or findings of the study | Such impacts are observable in the findings. |
| **DISCUSSION** |  |  |  |
| DISCUSSION Study findings, limitations, generalizability, and current knowledge | 26 | Report key findings, limitations, ethical or equity considerations not captured, and how these could impact patients, policy, or practice. | These are discussed in the discussion section. |
| **OTHER RELEVANT INFORMATION** |  |  |  |
| Source of funding | 27 | Describe how the study was funded and any role of the funder in the identification, design, conduct, and reporting of the analysis | This is stated at the end of the article. |
| Conflicts of interest | 28 | Report authors conflicts of interest according to journal or International Committee of Medical Journal Editors requirements. | This is stated at the end of the article. |

Husereau D, Drummond M, Augustovski F, de Bekker-Grob E, Briggs AH, Carswell C, Caulley L, Chaiyakunapruk N, Greenberg D, Loder E, Mauskopf J, Mullins CD, Petrou S, Pwu RF, Staniszewska S; CHEERS 2022 ISPOR Good Research Practices Task Force. Consolidated Health Economic Evaluation Reporting Standards 2022 (CHEERS 2022) Statement: Updated Reporting Guidance for Health Economic Evaluations. BMJ. 2022;376:e067975. The checklist is Open Access distributed in accordance with the terms of the Creative Commons Attribution (CC BY 4.0) license, which permits others to distribute, remix, adapt and build upon this work, for commercial use, provided the original work is properly cited. See: http://creativecommons.org/licenses/by/4.0/.
