# Supplementary figures and images for: Economic evaluation of hypertension screening in Iran using a Markov model
Source: PLoS One. 2025 Jul 22;20(7):e0303223. doi: 10.1371/journal.pone.0303223 (PMC12282904; doi:10.1371/journal.pone.0303223)

**The results of the deterministic sensitivity analysis of all strategies**


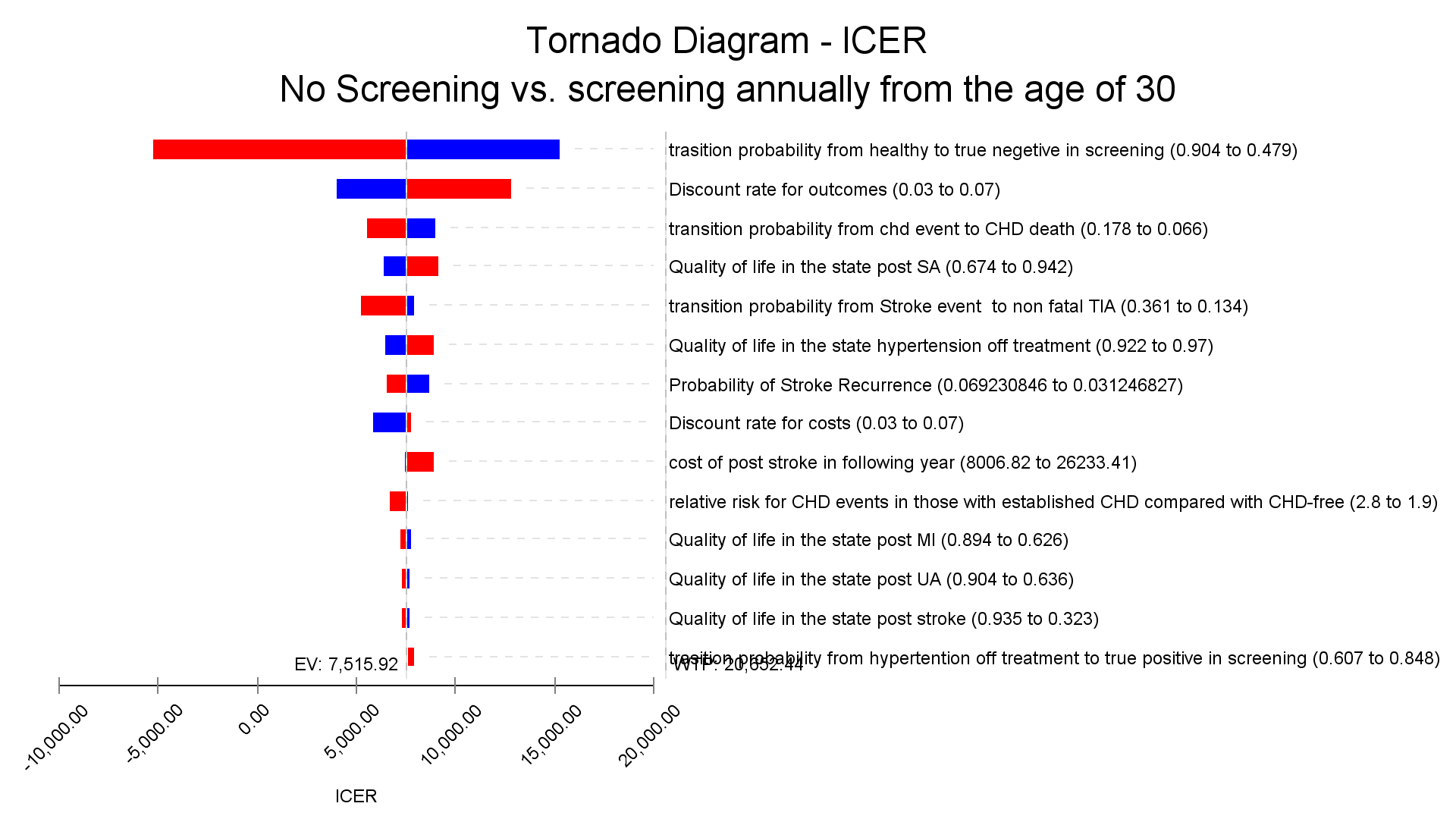


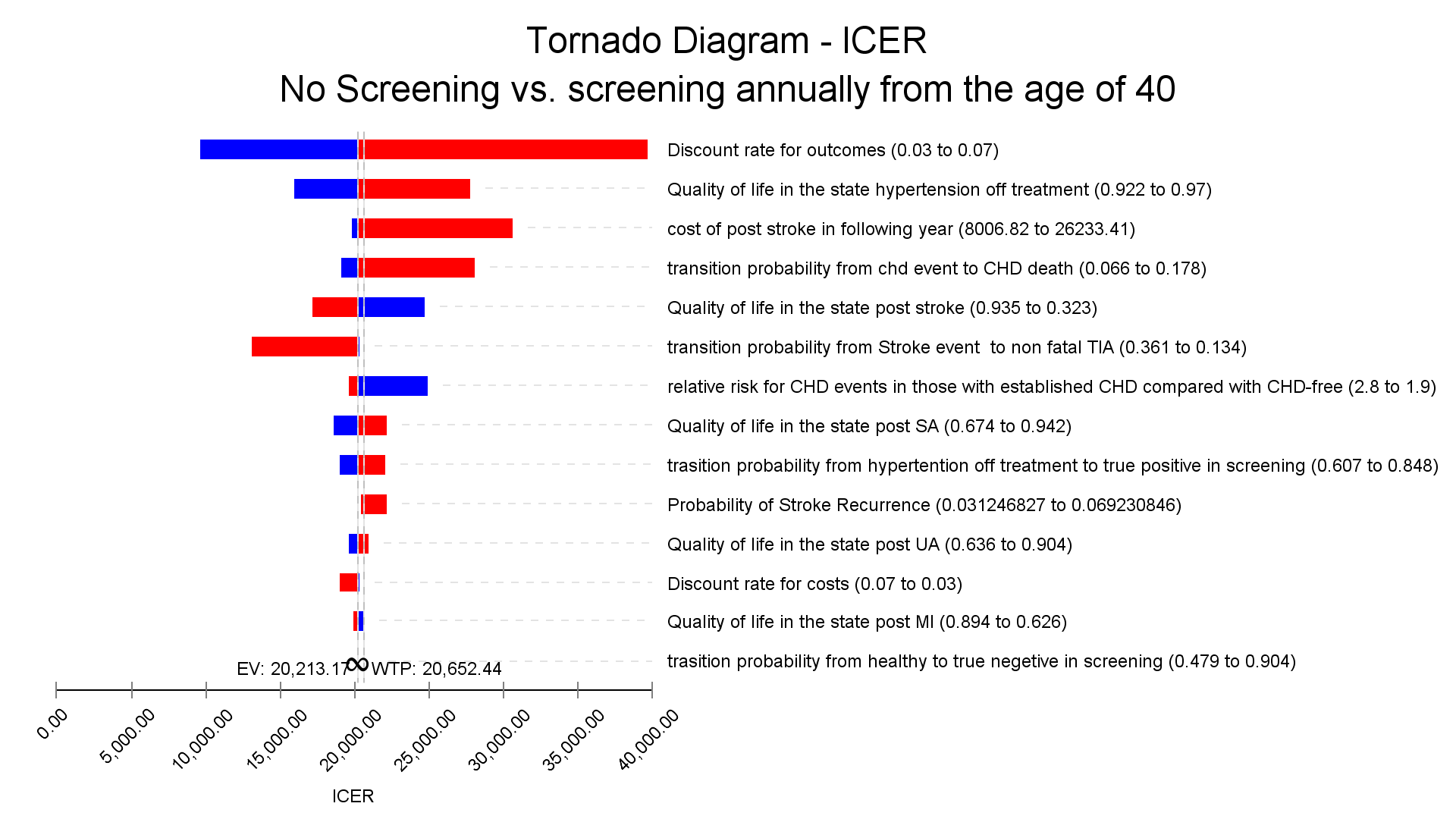


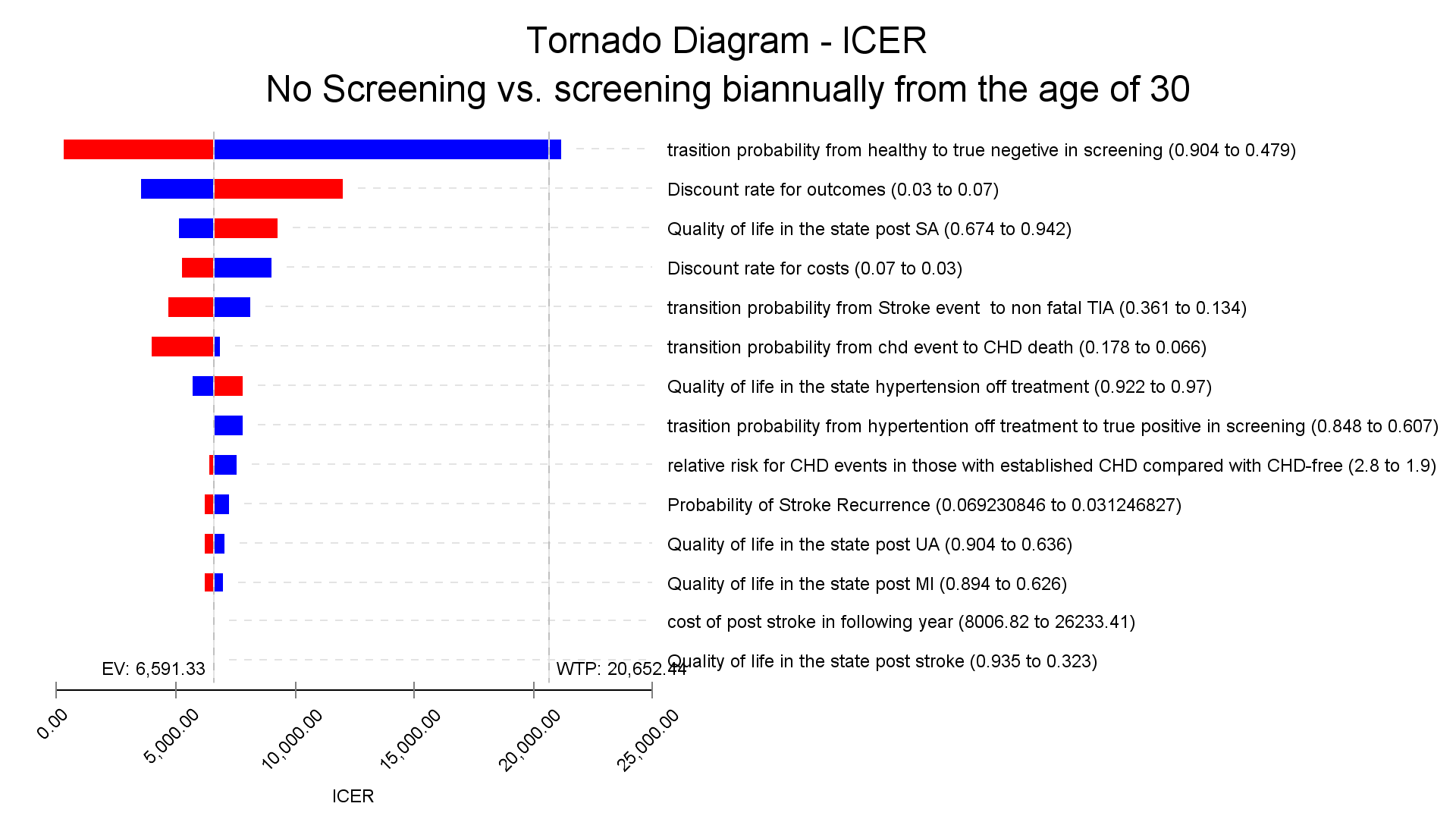


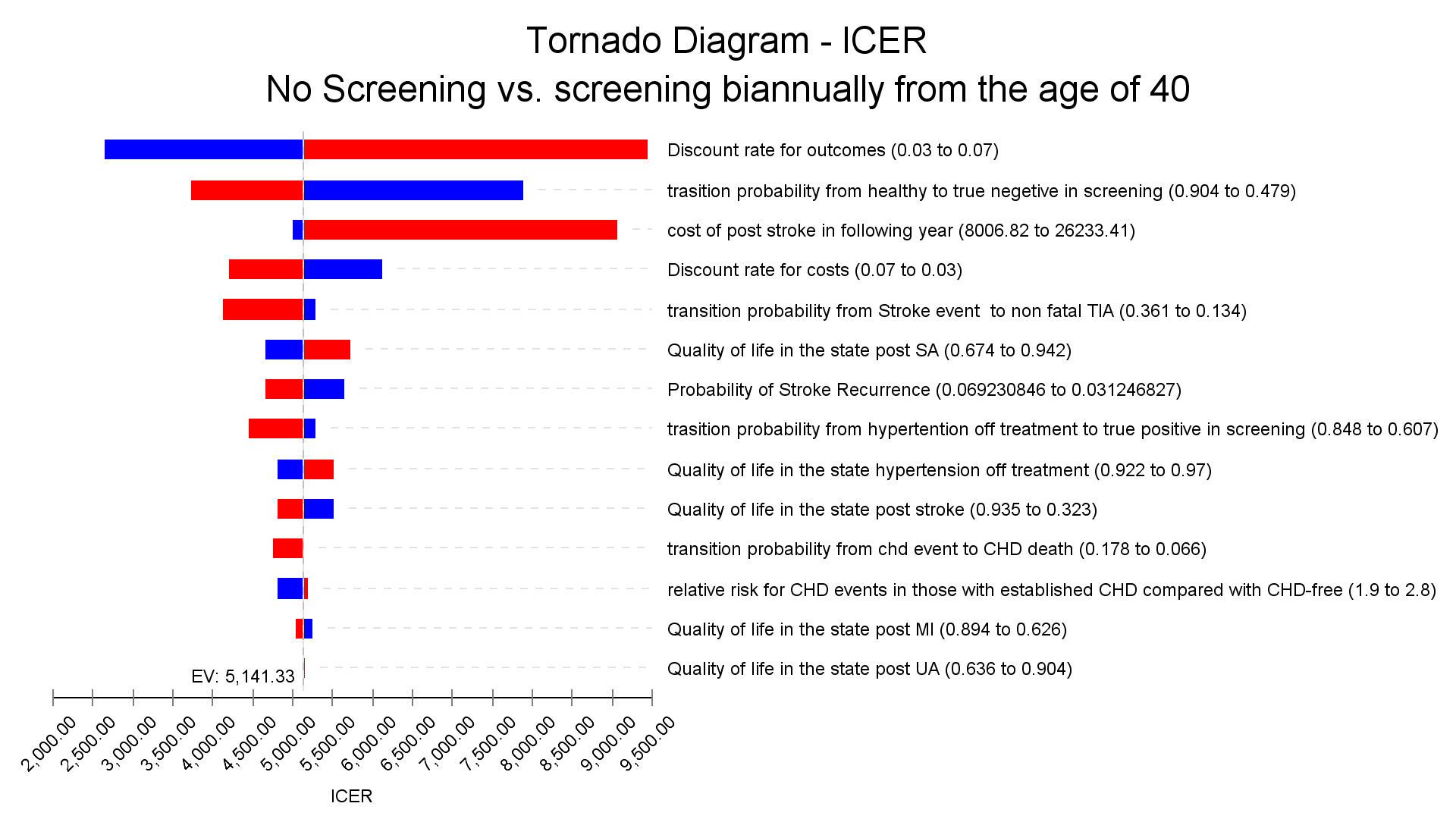


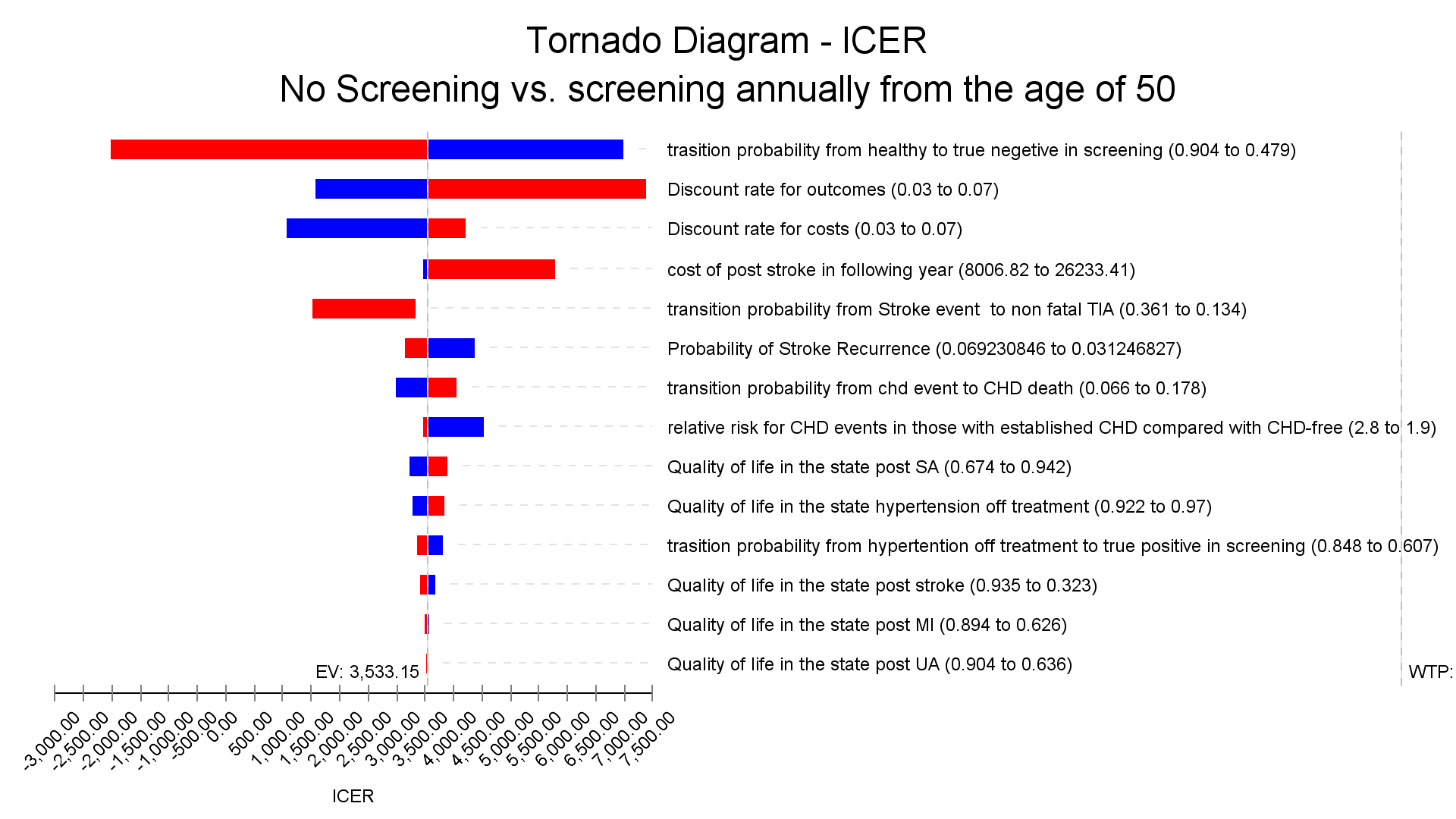


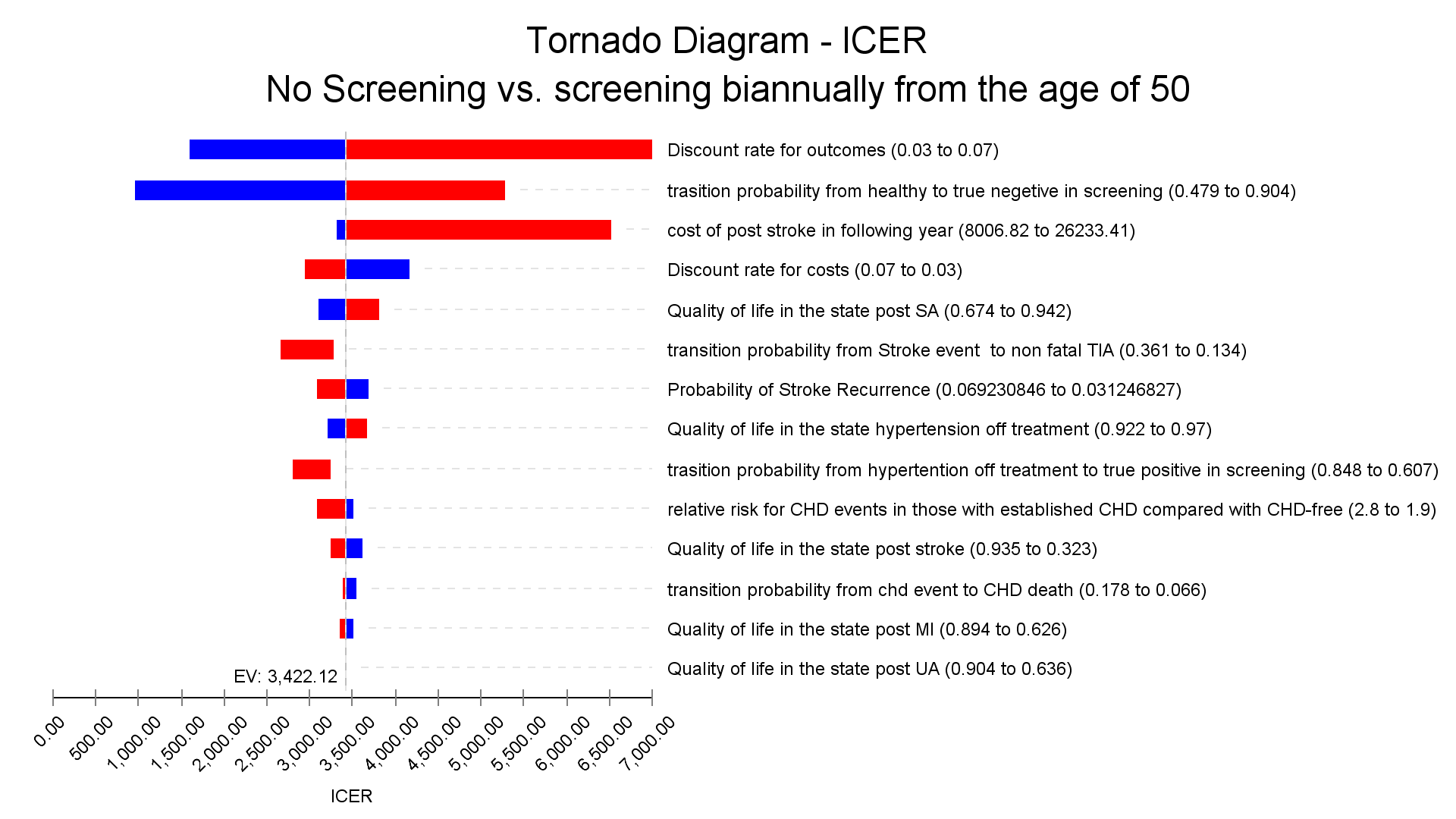


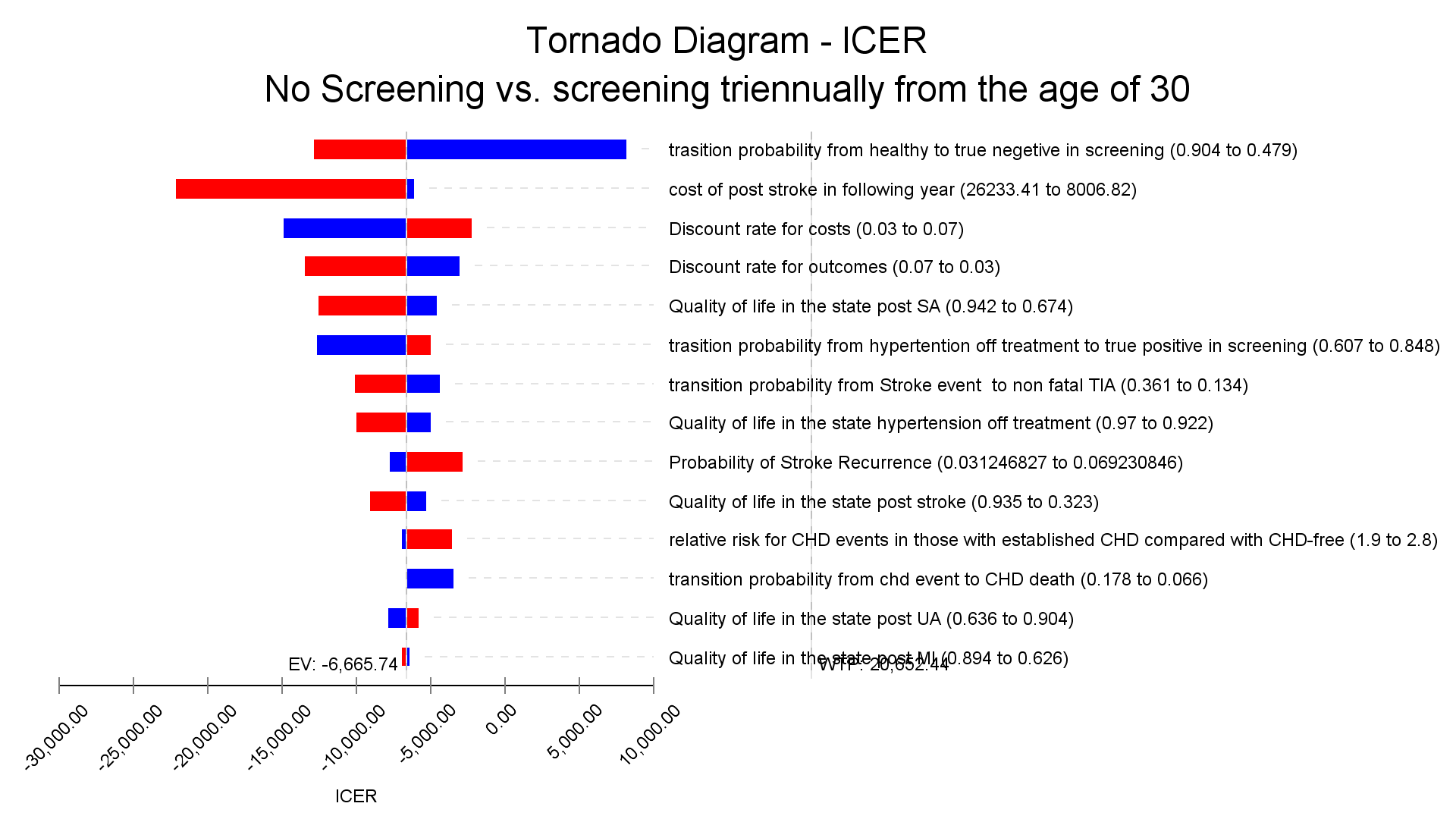


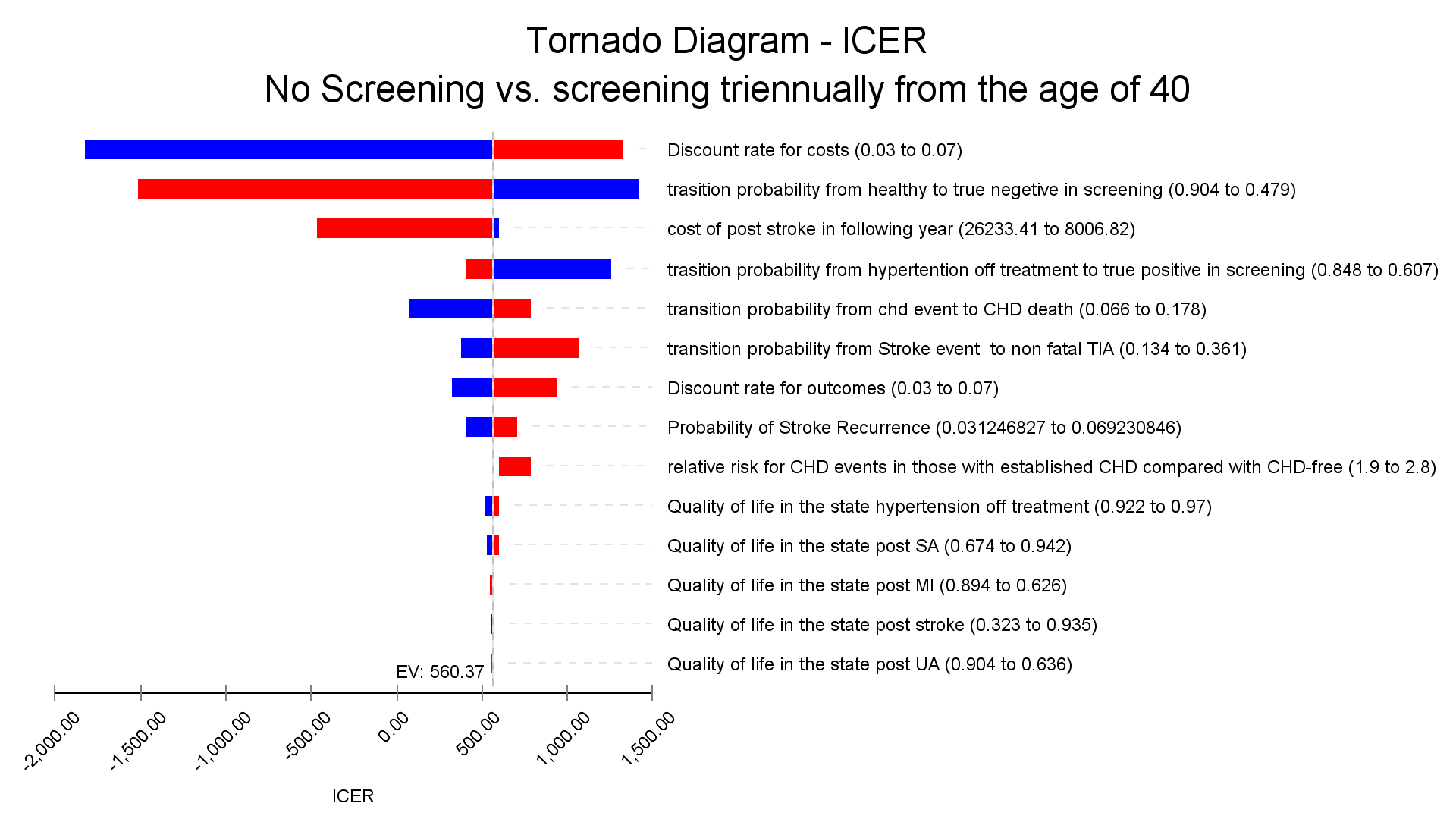


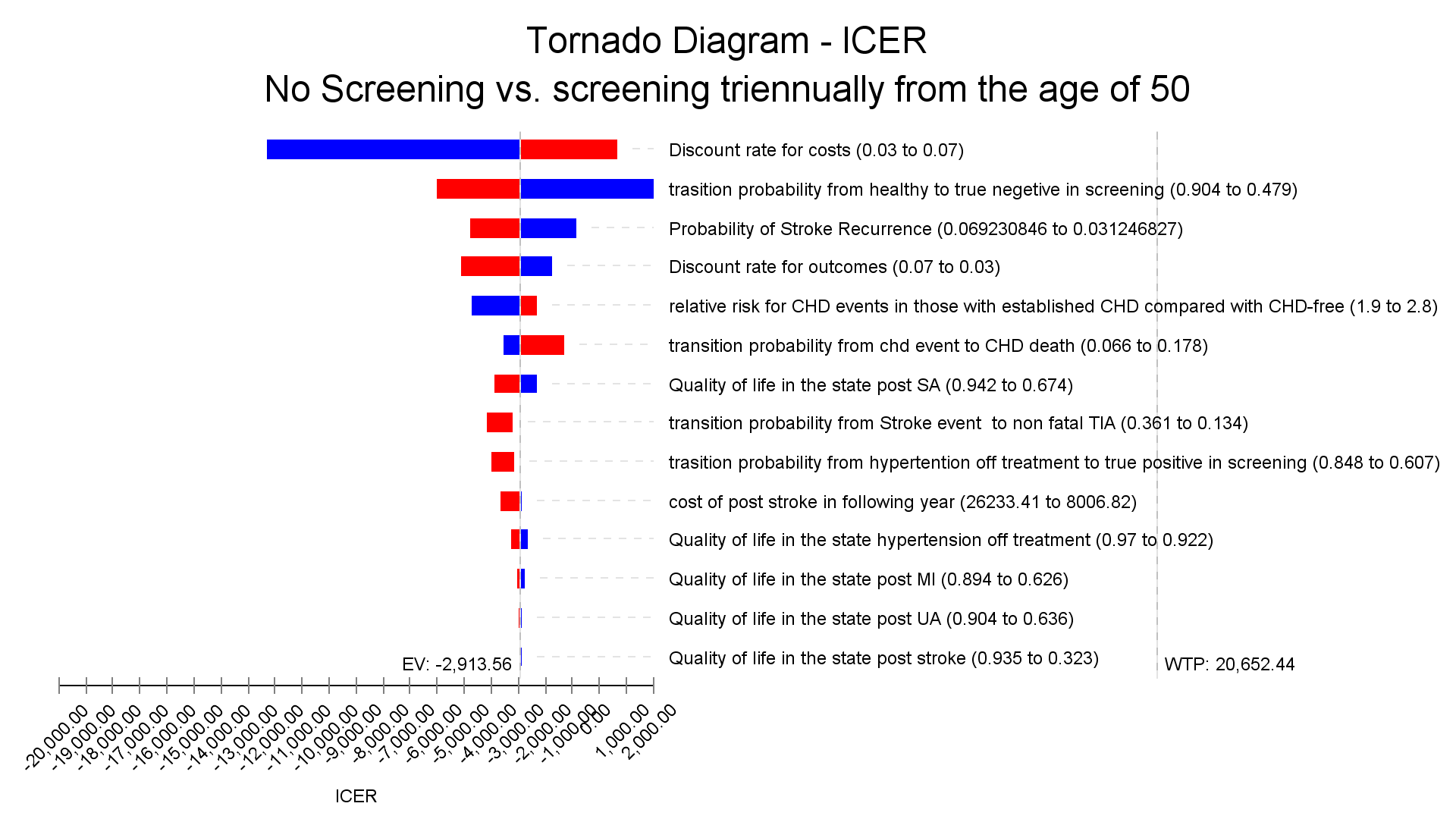

Supplement: S1 File — (DOCX) [file pone.0303223.s003.docx]
